# Mandible Reconstruction With Custom-Made Plates in Medication-Related Osteonecrosis of the Jaw—A Case Series

**DOI:** 10.1155/2024/9251185

**Published:** 2024-10-24

**Authors:** Eiji Mitate, Taichi Demura, Youta Yamauchi, Yasuhisa Sawai, Miho Hasumoto, Satoshi Wada, Hiroyuki Nakano, Noboru Demura

**Affiliations:** ^1^Department of Oral and Maxillofacial Surgery, Faculty of Medicine, Kanazawa Medical University, 1-1 Daigaku, Uchinada, Kahoku, Ishikawa 920-0293, Japan; ^2^Dentistry and Oral Surgery, Kouseiren Takaoka Hospital, 5-10 Eiraku-cho, Takaoka, Toyama 933-8555, Japan

**Keywords:** custom-made, mandible, reconstruction, selective laser melting, titanium plate

## Abstract

**Background**: Restoration of the mandibular region after segmental resection surgery is crucial for masticatory function and facial aesthetics. The precision of three-dimensional printers has advanced in recent years, enabling the development of fully customized reconstruction plates. In recent years, three-dimensional printing technology has been applied in the field of dental and oral surgery. Among these, the selective laser melting method has been developed. This case report was aimed at exploring the utility in restoring mandibular morphology.

**Cases:** Patients diagnosed with medication-related osteonecrosis of the jaw (MRONJ) in Oral and Maxillofacial Surgery at Kanazawa Medical University Hospital who underwent mandibular disarticulation and immediate completely customized reconstruction plate (COSMOFIX) were included. Case 1 involved a female in her 70s with MRONJ on the right side of the mandible. Case 2 involved a female who received bisphosphonates for extensive metastatic breast cancer, resulting in MRONJ affecting the bilateral mandible. Case 3 involved a woman who developed MRONJ on the right side of the mandible during alendronate treatment for osteoporosis. Completely customized reconstruction plates were used for reconstruction after segmental resections.

**Findings:** The three patients adapted to the remaining bone. The reconstructed and preoperative mandibular morphologies were similar.

**Conclusions**: In conclusion, the use of completely customized reconstruction plates obviates the need for bending and other adaptations, reduces surgical time, and improves postoperative aesthetics. Of significance, preparing completely customized reconstruction plate requires about 3 weeks; thus, careful case selection and scheduling are indispensable.

## 1. Introduction

The maxillofacial area is vital for chewing and talking as well as aesthetics, the deformation of which can impact social interactions. When faced with mandibular gingival carcinoma or an escalating prevalence of medication-related osteonecrosis of the jaw (MRONJ) leading to mandibular resection, it is crucial to reconstruct the mandibular morphology accurately to its preoperative state. Recent developments in three-dimensional (3D) printing technology have led to the creation of personalized reconstruction plates [1, 2].

This study was aimed at assessing the efficacy and problem of surgical procedures using a completely customized reconstruction plate (well-fitting plate for internal fixation; COSMOFIX, Osaka Yakin Kogyo Co. Ltd., Osaka, Japan).

## 2. Patients and Methods

Patients diagnosed with MRONJ at the Oral and Maxillofacial Surgery, Kanazawa Medical University Hospital, who underwent mandibular disarticulation and immediate reconstruction with COSMOFIX were included. Consent for this procedure is obtained from all patients.

COSMOFIX is a novel and custom-made selective laser melting (SLM) 3D-printed titanium plate. First, the patient's DICOM data is sent to the 3D printing manufacturer. The manufacturer constructs a 3D model of the mandibular bone using specialized analysis software. Then, a web conference is held between the doctors and the 3D printing manufacturer to confirm the resection area of the mandibular bone, the shape of the plate, the screw fixation positions, and other details. Once the final shape is determined, the plate is manufactured and delivered to the doctors in about 2 weeks. It is then sterilized and used in surgery.

## 3. Results

### 3.1. Case 1

**Figure 1:**
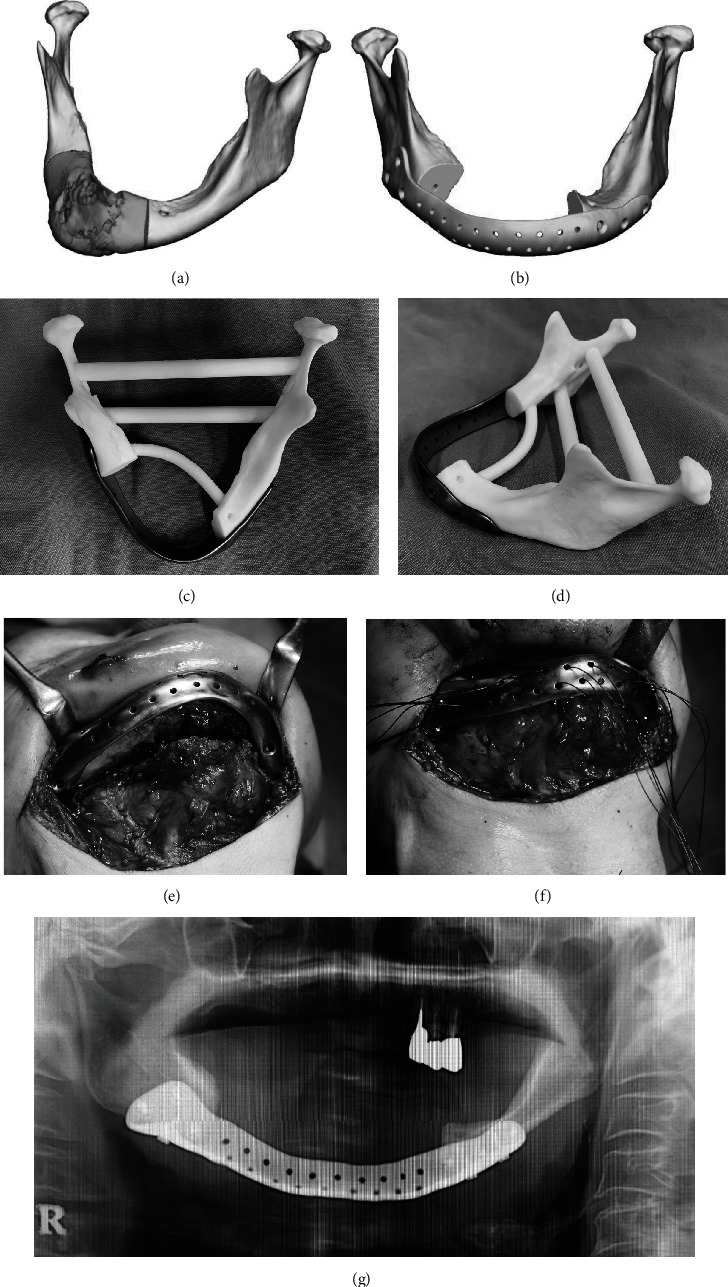
Case 1. (a) Range of mandibular resection (grey color area). (b) Three-dimensional (3D) design of the reconstruction plate after resection. (c) 3D model and reconstruction plate (occlusal view). (d) 3D model and reconstruction plate (view from the left side). (e) Verification of plate positioning. (f) After plate reconstruction. Traction of soft tissues with 1-0 nylon. (g) Postoperative panoramic image. The fit with the plate is excellent.

A 70-year-old woman (Figure 1) with a previous diagnosis of osteoporosis, right submandibular fistula, and a 5-year history of the use of sodium hydrate alendronate was referred to our department in January 2023. One year before her visit, the dental surgeon advised her to perform oral rinses alone.

During the initial examination, pantomography and computed tomography (CT) scans of the jaws revealed significant bone loss predominantly in the right bicuspid area of the mandible extending to the inferior margin (Figure 1(a)). DICOM data from CT images was shared with the manufacturer of the completely customized reconstruction plate (Figure 1(b)). During an online meeting with the manufacturer, we confirmed the region of the mandible resection, plate design, and screw positions to be fixed. The plate was manufactured upon confirmation of its strength (Figures 1(c) and 1(d)). The order process took 3 weeks.

Intraoperative results showed that the extent of resection and the screw fixation positions were validated by adapting the plate before resecting the mandibular area (Figure 1(e)). Good intraoperative maneuverability was observed due to preoperative decision-making on minimal resection. The genioglossus and geniohyoid muscle attachments were sutured to the plate to manage tongue root subsidence (Figure 1(f)). The operation lasted for a total of 171 min.

Postoperatively, the mandibular morphology was maintained with a favorable fit between the remaining bone and the plate (Figure 1(g)). No postoperative infections are seen.

### 3.2. Case 2

A female in her 80s was referred to us for MRONJ in 2021. In January 2019, she was diagnosed with left breast cancer that had metastasized to her axillary lymph nodes, liver, and bones. She received treatment consisting of pertuzumab and trastuzumab. After treatment with zoledronic acid hydrate, MRONJ developed in the left mandible. In 2022, we performed mandibular marginal resection and inserted a plate that could be bent during surgery. However, the plate was later extracted due to a postoperative infection. Despite its removal, the discharge of pus continued.

**Figure 2:**
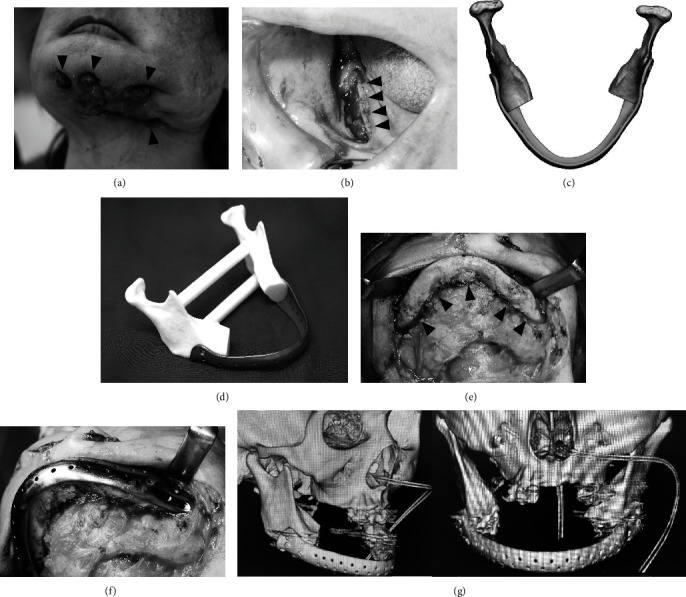
Case 2. (a) Preoperative extraoral photograph. Four large fistulas are observed extending from the chin to the left mandible (arrowhead). (b) Preoperative intraoral photograph. Mandibular bone exposure is observed in the right lower gingiva (arrowhead). (c) Three-dimensional (3D) design of the reconstruction plate. (d) 3D model and reconstruction plate. (e) Intraoperative photograph. The mandible bone is clearly indicated (arrowhead). (f) Confirming the plate on the mandible bone. (g) Postoperative computed tomography. The adaptation between the plate and bone was acceptable.

CT after readmission revealed substantial bone resorption in the left mandible extending to the inferior margin. The left mandible exhibited multiple fistulas (Figure 2(a)), while osteoclastic activity was noted on the right mandible (Figure 2(b)). A suspected pathological fracture was observed in the left mandible. To address this, partial resection of the mandible and reconstruction with a completely customized reconstruction plate were planned. The plate was manufactured in the same way as in Case 1 (Figure 2(d)).

The overall condition of the patient was stable enough to undergo surgery, and chemotherapy for breast cancer was paused for 4 weeks prior to the operation.

Intraoperatively, after the fistulas were eliminated, a skin incision was performed. The mandible was clarified to the minimum extent necessary (Figure 2(c)). The plate was adapted before the area was sectioned to confirm the resection extent and screw fixation positions (Figures 2(e) and 2(f)). Intraoperative maneuverability was preferred. To maintain tongue movement, the genioglossus muscle and geniohyoid muscle were sutured to the plate using 1-0 nylon. The operative time was 312 min.

Postoperatively, the mandibular morphology was preserved and the precision between the remaining bone and plate was satisfactory (Figure 2(g)). No postoperative infections are seen. The patient is followed up with attention to signs of plate infection.

### 3.3. Case 3

**Figure 3:**
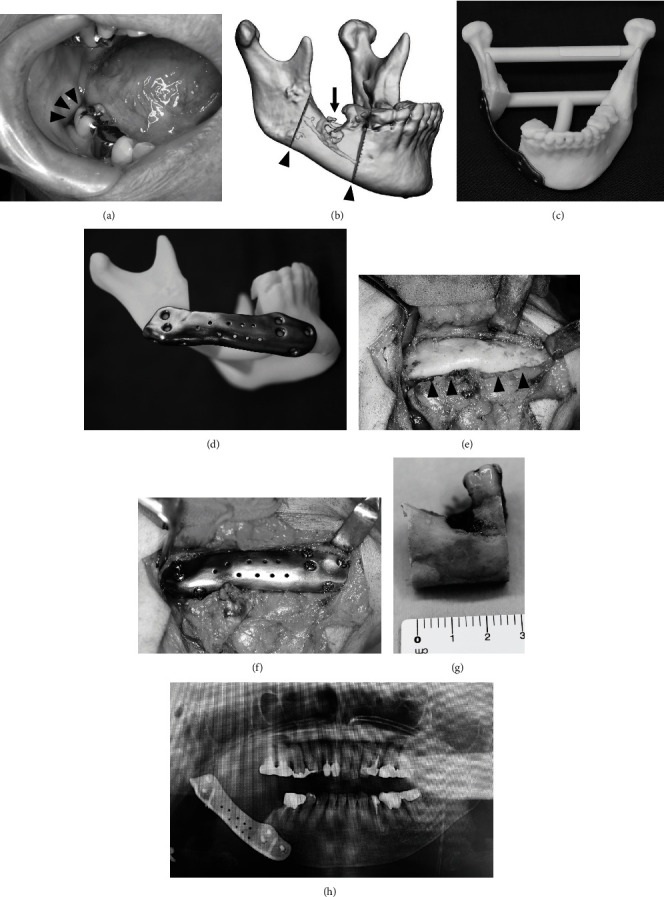
Case 3. (a) A small fistula was observed in the right lower molar (arrowhead). (b) Planning image of the osteotomy line (arrowhead). Resorption of the mandible is observed (arrow). (c) Three-dimensional (3D) model and reconstruction plate (frontal view). (d) 3D model and reconstruction plate (right lateral view). (e) Clarification of the right mandible bone (arrowhead). (f) After plate reconstruction. (g) Resected bone. (h) Postoperative panoramic image. The adaptation between the plate and bone was preferable.

In May 2023, our department received a referral for a female patient in her 70s with MRONJ affecting the right mandible. She was diagnosed with osteoporosis in 2020 and treated with alendronic acid. In March 2023, the patient sought care at a local dental clinic for inflammation of the right mandible and was subsequently referred to our department. During the initial examination, panoramic and CT images showed cemental dysplasia in the region of the right second molar of the mandible accompanied by bone resorption (Figure 3(a)). Due to concerns about the potential for fracture based on the remaining bone volume during marginal resection, mandibular resection was planned. To address the soft tissue and bone defect, we planned to apply the cervical island flap and a completely custom reconstruction plate (Figures 3(c) and 3(d)).

Intraoperatively, the right mandible was clearly visible after a 40 × 70 mm cervical island flap was raised (Figure 3(e)). Following an oral mucosal incision, the plate was properly placed prior to resection. Plate fixation was performed (Figure 3(f)), and the flap was rotated from the lingual side to fill the bone and gingival soft tissue defects. The surgical procedures lasted for 201 min. The plate adaptation was confirmed as being almost identical to the preoperative plan (Figure 3(h)). No postoperative infections are seen.

## 4. Discussion

Various difficulties exist in the use of reconstruction plates [3]. In mandibular reconstruction, it is important to determine to what extent the reconstructive plate maintains facial morphology. Conventional commercially available reconstructive plates are flat and straight and require arduous and extended manual bending to match the bone morphology. Intraoperative bending prolongs the operational time and involves a heavy burden on the patient. The bending time depends on the surgeon's expertise.

To shorten the operating time, methods such as using a 3D printer to produce a mandible model from DICOM data and bending the plate in advance have been reported [4–6]. In either case, minimal bending is required to prevent postoperative plate fractures.

Plate thickness and size should be as thin and small as possible since it affects facial morphology. However, occlusal forces must be considered, including the number of remaining teeth, whether soft tissue reconstruction is performed after mandibular resection, and whether plate reconstruction is performed. Depending on the balance of these factors, plate fracture may occur [7].

Despite these considerations, postoperative plate exposure may still occur [8]. In Case 2, the patient is followed up with attention to signs of plate infection.

Considering the issues, a product that is less influenced by surgeon experience, does not require intraoperative plate bending, conforms firmly to the individual patient's mandibular morphology, and features a shape that is less likely to be exposed is desirable.

In recent years, developments have occurred in mandibular reconstruction, including the creation of preformed plates that require less bending to conform to the patient's mandibular morphology [9, 10]. Additionally, the fibula can be formed according to a preoperatively designed template [11], and custom titanium plates are now available that can replicate the patient's mandibular shape using 3D printing technology [1–3, 12].

SLM is an advanced additive manufacturing (3D printing) technology used to produce high-precision, high-density metal parts. The process of SLM is five steps. First, a digital 3D model of the part is created using computer-aided design (CAD) software. This model is then sliced into thin layers. Second, a thin layer of metal powder (e.g., titanium, aluminum, and stainless steel) is spread over the build platform. Third, a high-power laser selectively scans and melts the metal powder according to the design of the sliced layer. The laser fully melts the powder, causing it to fuse and solidify into a dense, solid layer. Fourth, the build platform is lowered slightly, and a new layer of metal powder is spread over the previous one. The laser then scans and melts this new layer, fusing it to the layer beneath. This process is repeated layer by layer until the entire part is built. Finally, once the build is complete, the part is removed from the powder bed and any excess powder is cleaned off. The part may then undergo further processing, such as heat treatment, machining, or surface finishing, to achieve the desired mechanical properties and finish.

SLM plates have many advantages [13]. SLM plates can produce highly complex and intricate designs that would be difficult or impossible to achieve with traditional manufacturing methods. SLM plates use only the material necessary for the part, with minimal waste, and produce plates with high density and excellent mechanical properties comparable to those made by traditional methods. In the field of oral and maxillofacial surgery, SLM technology is now being applied not only to mandibular reconstruction but also to temporomandibular joints and dental implants. It holds the potential for even wider use in the future [14, 15].

The limitations of using a custom plate during surgery include a 3-week modelling period and the inability to expand the resection area once modelling has begun. A preoperative imaging assessment is critical for determining the resection extent. Due to the longer delivery times, the use of custom plates is preferred for noncritical treatments and may be more problematic in patients with malignant tumors.

For standard and custom plates, the occlusal forces must not exceed the allowable stresses of the plate and screw [16]. Therefore, extended postoperative monitoring is essential regardless of the type of plate used.

## Data Availability

Due to the nature of this research, participants of this study did not agree for their data to be shared publicly, so supporting data is not available.
